# CXCR2 Inhibition – a novel approach to treating CoronAry heart DiseAse (CICADA): study protocol for a randomised controlled trial

**DOI:** 10.1186/s13063-017-2210-2

**Published:** 2017-10-11

**Authors:** Jubin P. Joseph, Eliana Reyes, Josephine Guzman, Jim O’Doherty, Hannah McConkey, Satpal Arri, Rahul Kakkar, Nicholas Beckley, Abdel Douiri, Sally F. Barrington, Simon R. Redwood, Albert Ferro

**Affiliations:** 1 British Heart Foundation Centre of Excellence, The Rayne Institute, St. Thomas’ Hospital, London, SE1 7EH UK; 2 PET Centre, St. Thomas’ Hospital, Westminster Bridge Road, London, SE1 7EH UK; 3 Scientific Partnering & Alliances, Innovative Medicines and Early Development Biotech Unit, AstraZeneca, 35 Gatehouse Drive, Waltham, Massachusetts 02451 USA; 4 Department of Primary Care and Public Health Sciences, King’s College London, London, SE1 1UL UK; 5 Department of Cardiology, St. Thomas’ Hospital, Westminster Bridge Road, London, SE1 7EH UK; 6 Cardiovascular Clinical Pharmacology, British Heart Foundation Centre of Research Excellence, Cardiovascular Division, King’s College London, London, UK

**Keywords:** Atherosclerosis, CXCR2, Neutrophil function, Coronary physiology, Coronary flow reserve

## Abstract

**Background:**

There is emerging evidence of the central role of neutrophils in both atherosclerotic plaque formation and rupture. Patients with lower neutrophil counts following acute coronary syndromes tend to have a greater coronary flow reserve, which is a strong predictor of long-term cardiovascular health. But so far, no data are available regarding the impact of neutrophil inhibition on cardiovascular clinical or surrogate endpoints. Therefore, the aim of this study is to investigate the effects of AZD5069, a cysteine-X-cysteine chemokine receptor 2 (CXCR2) inhibitor, on coronary flow reserve and coronary structure and function in patients with coronary artery disease.

**Methods/Design:**

Ninety subjects with coronary artery disease undergoing percutaneous coronary intervention will be included in this investigator-driven, randomised, placebo-controlled, double-blind, phase IIa, single-centre study. Participants will be randomised to receive either AZD5069 (40 mg) administered orally twice daily or placebo for 24 weeks. Change in coronary flow reserve as determined by ^13^N-ammonia positron emission tomography-computed tomography will be the primary outcome. Change in the inflammatory component of coronary plaque structure and the backward expansion wave, an invasive coronary physiological measure of diastolic function, will be assessed as secondary outcomes.

**Discussion:**

Cardiovascular surrogate parameters, such as coronary flow reserve, may provide insights into the potential mechanisms of the cardiovascular effects of CXCR2 inhibitors. Currently, ongoing trials do not specifically focus on neutrophil function as a target of intervention, and we therefore believe that our study will contribute to a better understanding of the role of neutrophil-mediated inflammation in coronary artery disease.

**Trial registration:**

EudraCT, 2016-000775-24. Registered on 22 July 2016.

International Standard Randomised Controlled Trial Number, ISRCTN48328178. Registered on 25 February 2016.

**Electronic supplementary material:**

The online version of this article (doi:10.1186/s13063-017-2210-2) contains supplementary material, which is available to authorized users.

## Background

Atherosclerosis, the principal cause of myocardial infarction and stroke, is a progressive inflammatory disease characterised by the accumulation of lipids and fibrous elements in large arteries [1]. Being an inflammatory disease, atherosclerosis involves recruitment of leucocytes (predominantly neutrophils and monocytes) to sites of vascular injury. Typically, this is triggered by the accumulation of oxidised low-density lipoprotein (LDL) within the intima, which stimulates endothelial cells to express an atherosclerotic phenotype and leads to the adherence of leucocytes on their surface. These inflammatory cells transmigrate into the intima, where monocytes proliferate and differentiate into macrophages which take up oxidised LDL, forming foam cells [1].

Atherosclerotic plaques exhibit two major phenotypes: (1) stable plaques, characterised by a thick fibrous cap separating a relatively small lipid core from the lumen, which are associated with a low risk of thrombotic complications; and (2) unstable plaques, characterised by a large lipid core covered by a thin fibrous cap prone to rupture and thrombus formation and associated with a higher risk of thrombotic complications [2, 3]. Leucocytes—in particular neutrophils and monocytes—are involved both in atherogenesis and in plaque destabilisation, the latter especially so for neutrophils [4, 5]. Specifically, neutrophils are over-represented in the vasculature of patients with unstable atherosclerotic plaque [4] and are associated with histologic features of plaque vulnerability [5, 6]. In addition to these established structural changes, a high-fat meal has been shown to elicit a mild neutrophilia along with endothelial dysfunction [7]. In recent studies, colchicine, a known neutrophil chemotaxis inhibitor, has been shown to reduce vascular events in patients with coronary heart disease [8].

The control of migration of neutrophils into coronary plaque is not fully understood, but it is thought to involve cysteine-X-cysteine chemokine receptor 2 (CXCR2) [9]. This G protein-coupled receptor binds interleukin-8 (IL-8) with high affinity together with chemokine (C-X-C motif) ligands 1, 2, 3 and 5 (CXCL1, CXCL2, CXCL3 and CXCL5, respectively). CXCR2 is expressed at a high level in neutrophils and plays a crucial role in mediating neutrophil migration, such that its inhibition greatly decreases neutrophil recruitment to sites of inflammation [10, 11]. In previous studies, AZD5069, a specific CXCR2 antagonist, was found to be potent in inhibiting calcium flux, chemotaxis and CD11b expression on human neutrophils in vitro in response to the CXCR2 ligands IL-8 and growth related oncogene-α [12]. In a rodent lipopolysaccharide (LPS) challenge model of pulmonary inflammation, AZD5069 reduced LPS-induced neutrophilia in a dose-dependent manner in both bronchial fluid and serum [13].

Because neutrophil recruitment into atherosclerotic plaques plays a crucial role in plaque destabilisation and vascular inflammation, it is possible that CXCR2 inhibition will give rise to improved coronary endothelial function, which will be reflected in an improvement in total coronary flow reserve (CFR). CFR is an integrated measure of coronary artery status encompassing both macro- and microvascular function, and it has prognostic value over and above other markers of cardiovascular risk [14]. Therefore, improvement in CFR with CXCR2 inhibition would be expected to translate into prognostic benefits clinically in long-term follow-up.

In addition to the effects on coronary flow dynamics, decreased neutrophil recruitment may also translate into a reduction in the high-risk morphologic features of coronary plaque (visualised by virtual histology intravascular ultrasound [VH-IVUS] as a diminution in size of the necrotic core of lesions). Additionally, there is evidence that neutrophil infiltration is involved in the pathophysiology of restenosis following coronary angioplasty [15, 16].

Therefore, in the CXCR2 Inhibition – a novel approach to treating CoronAry heart DiseAse (CICADA) study, we aim to perform a randomised, placebo-controlled, double-blind trial to investigate the effect of the CXCR2 inhibitor AZD5069 on CFR and coronary plaque morphology. This will help to improve understanding of the cardiovascular effects of CXCR2 inhibition in patients with coronary artery disease (CAD).

## Methods

### Design

This is a prospective, randomised, placebo-controlled, double-blind, phase IIa, single-centre study to evaluate the effect of the CXCR2 antagonist AZD5069 40 mg twice daily on cardiovascular surrogate measures in patients with CAD undergoing percutaneous coronary intervention (PCI). The trial was approved by the South Central - Berkshire B Research Ethics Committee (16/SC/0478) and is to be conducted at the Department of Cardiology, St. Thomas’ Hospital, London, UK. The study is co-sponsored by King’s College London and Guy’s and St. Thomas’ NHS Foundation Trust.

**Fig. 1:**
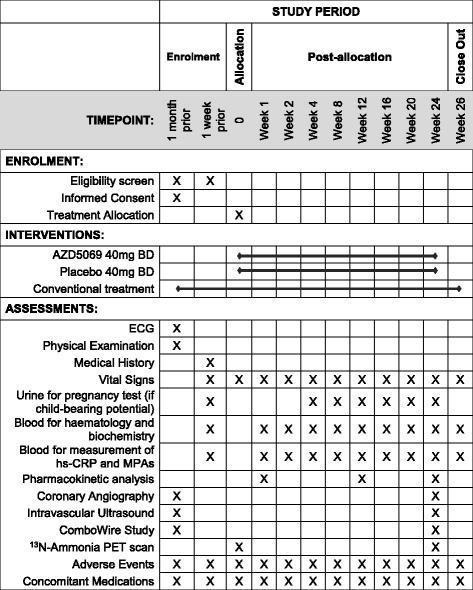
Standard Protocol Items: Recommendations for Interventional Trials (SPIRIT) figure for the CXCR2 Inhibition – a novel approach to treating CoronAry heart DiseAse (CICADA) trial, outlining schedule for enrolment, intervention and assessments. *ECG* Electrocardiogram, *hs-CRP* High-sensitivity C-reactive protein, *MPA* Monocyte-platelet aggregates, *PET* Positron emission tomography

All participants will be asked to provide written informed consent before entering the study. Clinical trial authorisation has been obtained from the Medicines and Healthcare Products Regulatory Agency (MHRA) (EudraCT, 2016-000775-24). A certificate for the administration of radioactive medicinal products has been granted by the UK Administration of Radioactive Substances Advisory Committee (ARSAC) (RPC 261/3492/35652). Important protocol modifications will be reported to the South Central - Berkshire B Research Ethics Committee and, if necessary, to the MHRA and ARSAC. This study follows the international recommendations for interventional trials (*see* Fig. 1 and the Standard Protocol Items: Recommendations for Interventional Trials [SPIRIT] checklist in Additional file 1).

### Primary objective

The primary objective of this trial is to determine, in patients undergoing PCI for atherosclerotic coronary disease, if 24 weeks of CXCR2 inhibition therapy will give rise to improvement in CFR as measured by ^13^N-ammonia positron emission tomography-computed tomography (PET-CT) compared with placebo.

### Secondary objective

The secondary objectives of the study are to ascertain, in patients undergoing PCI for atherosclerotic coronary disease, if 24 weeks of CXCR2 inhibition therapy will result in a reduction in the inflammatory component of coronary plaque, a decrease in the degree of in-stent restenosis and improvements in coronary microvascular function compared with placebo.

### Inclusion and exclusion criteria

Participants in the trial will be aged ≥ 18 years, have angiographically proven coronary heart disease, be undergoing native-vessel PCI, and have a persistent neutrophil count > 4.0 × 10^9^/L. Otherwise, they will be receiving the standard of care for ischaemic heart disease (including appropriate antiplatelet therapy, statin, and/or antihypertensives as clinically indicated).

The exclusion criteria are as follows: acute ST-elevation myocardial infarction, prior cardiovascular surgery, known active or recent infection, evidence of tuberculosis, known immunocompromised state or history of organ transplantation, known major organ dysfunction or other significant co-morbidity, use of a cytochrome P450 3A4 inhibitor and inducers, alanine aminotransferase or aspartate aminotransferase level ≥ 2.5 times the upper limit of normal, QTc > 450 milliseconds for males and >470 milliseconds for females, pregnancy or breastfeeding during the study, women of childbearing potential not using a highly effective method of contraception, and unwilling or unable to give informed consent.

### Eligibility and recruitment

The study population will consist of 90 subjects with CAD undergoing PCI. Subjects are identified from inpatient admissions and outpatient angioplasty waiting lists at St. Thomas’ Hospital, London, UK. The direct care team staff will inform patients about the possibility of being enrolled in this study. No study-related procedures are undertaken before obtaining informed consent. A member of the study team will explain the study procedures in detail and provide the participant with a patient information sheet. After a minimum of 2 h, but ideally more than 24 h, the participant will be asked about their willingness to participate in this research study, and any questions will be clarified. After informed consent is obtained, participants will be given a copy of the informed consent form to sign and assigned a screening identification number.

### Screening

Screening of participants consists of the evaluation of inclusion and exclusion criteria. After informed consent is obtained by a member of the trial team, an electronic case report form (eCRF) is created for potential participants, including information about medical history, physical examination and their current medications. Women of childbearing potential undergo urine pregnancy testing.

### Participant timeline

If eligible, subjects are scheduled for the following study visits (Fig. 1). Patients undergo invasive coronary angiography with PCI as per normal clinical care, but they will also undergo IVUS to provide greyscale and virtual histology of all major coronary arteries. Patients with suitable coronary anatomy will be invited to have invasive assessment of coronary and microvascular physiology under adenosine-induced hyperaemia (using a ComboWire; Volcano Corp., Rancho Cordova, CA, USA).

Four weeks later, subjects will re-attend the cardiology department, at which time a full history and physical examination will be performed and inclusion criteria confirmed. Assessments will include a 12-lead electrocardiogram, as well as blood tests (fasted) for blood count and biochemistry (renal, liver, lipid, glycaemic and thyroid profiles) and high-sensitivity C-reactive protein (hs-CRP) and homocysteine levels. Screening blood tests for disorders which may exclude patients from the study will be drawn as indicated (e.g., serum hepatitis B surface antigen, hepatitis C antibody, and HIV testing if prompted by history). Additionally, an aliquot of blood will be taken for measurement of circulating monocyte-platelet aggregates (MPAs, a highly sensitive and reproducible index of degree of platelet activation) by flow cytometry. In the case of women of childbearing potential, pregnancy will be excluded by performing a pregnancy test on a spot urine. Once eligibility has been confirmed, ^13^N-ammonia PET-CT cardiac scans will be obtained at rest and during adenosine-induced hyperaemia to measure CFR. The CT scan will be obtained prior to the PET scan for attenuation correction at rest and stress, and prior to the rest scan, the CT scan will also be used to measure calcium score.

**Fig. 2:**
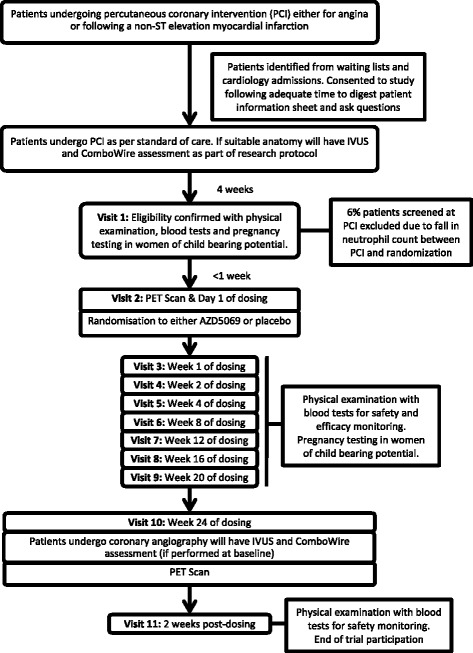
Flowchart of CXCR2 Inhibition – a novel approach to treating CoronAry heart DiseAse (CICADA) trial protocol. Flowchart depicts the study process and patient visit details for enrolled candidates in the CICADA study. *PCI* Percutaneous coronary intervention, *PET* Positron emission tomography, *IVUS* Intravascular ultrasound

After randomisation, subjects will be allocated 2:1 to receive either AZD5069 or matched placebo, orally, for the succeeding 24 weeks. At different time points (weeks 1, 2, 4, 8, 12, 16, 20 and 24), patients will attend for repeat visits, at which time they will undergo a physical examination and have a blood sample taken for the purpose of determining safety and efficacy biomarkers. At weeks 1, 12 and 24, patients will attend prior to their morning dose, and additional blood samples will be taken for the purpose of measuring plasma drug levels. At weeks 4, 8, 12, 16, 20 and 24, spot urine samples will be obtained from women of childbearing potential in order to exclude pregnancy; if they have a positive test result at any point, they will be immediately withdrawn from the study. At week 24, a repeat ^13^N-ammonia PET scan will be obtained to assess change from baseline in total CFR, and at the same time point, repeat coronary angiography together with IVUS will be performed to assess change in plaque composition and degree of restenosis. Those patients who initially had a ComboWire assessment will be invited to have a repeat assessment at the time of their repeat coronary angiography. A flow diagram of the protocol is shown in Fig. 2.

### Outcome measurements

The primary outcome is the change in total CFR from baseline, as measured by ^13^N-ammonia PET, before and after 24 weeks of treatment with AZD5069 or matched placebo. The secondary endpoints are as follows:

1. Change in plaque composition, as measured by VH-IVUS, before and after 24 weeks of treatment with AZD5069 or matched placebo
2. Degree of in-stent restenosis, as measured by IVUS, before and after 24 weeks of treatment with AZD5069 or matched placebo
3. Change in the magnitude of the backward expansion wave (BEW), as measured by ComboWire assessment, before and after 24 weeks of treatment with AZD5069 or matched placebo

### Randomisation and blinding

Participants will be randomised to one of the two study arms (AZD5069 versus placebo) in a 2:1 ratio in real time by using the web-based randomisation service provided by the King’s Clinical Trials Unit. The randomisation system is programmed to use block randomisation with randomly selected block sizes. The randomisation system sends unblinded treatment allocation codes to the pharmacy department at Guy’s and St. Thomas’ NHS Foundation Trust and the Emergency Scientific and Medical Services (ESMS).

The pharmacy department will use treatment allocation to dispense appropriate study medication or matched placebo. The labels of the study medication display the name of the trial, the name of the investigational medicinal product and contact details of the principal investigator.

A 24-h emergency code break and medical information service will be provided by ESMS staff, who are not involved as study investigators. Each randomised subject will be provided with a card detailing code break telephone numbers and emergency contact details. Subjects will be requested to carry this card with them at all times whilst participating in the trial.

### Interventions

The subjects will receive either AZD5069 40 mg or matched placebo tablets administered orally twice daily for 24 weeks. The drug and placebo are manufactured by AstraZeneca (London, UK). The drug product is presented as plain, round, beige, film-coated tablets packaged in bottles. The tablets contain mannitol, microcrystalline cellulose, croscarmellose sodium, sodium lauryl sulphate, sodium stearyl fumarate, polyvinyl alcohol, titanium dioxide, polyethylene glycol, talc, iron oxide yellow, iron oxide red and black iron oxide. Matching placebo tablets are identical in appearance and tasteless. The pharmacy department at Guy’s and St. Thomas’ NHS Foundation Trust packs the medication as 2- to 4-week supplies for study participants and labels the study medication per current regulatory requirements.

At each visit, pill counts will be conducted to verify medication adherence. Significant non-adherence is defined as missing 20% or more doses of trial medication during the treatment period. In the case of study medication non-compliance, participants will continue to be followed to their last visit. These data would not be part of the per-protocol analysis but would form part of an intention-to-treat analysis.

Any investigational medicinal product, dispensed and unused or not dispensed, will be disposed of by the pharmacy department at Guy’s and St. Thomas’ NHS Foundation Trust. Withdrawal of consent and major protocol violations will lead to an early study termination for the subject.

### Physical measurements

Anthropometric measurements are performed on each participant at each study visit. Weight is measured with the patient standing and then registered after rounding it to the nearest 500 g. Height is measured using a metric tape with the patient standing against a wall, and the value is marked by a ruler placed horizontally on the vertex of the patient’s head. Blood pressure is taken using an automated sphygmomanometer after a 5-minute rest in sedentary body position. Core temperature is taken using a tympanic membrane thermometer.

### Procedures for assessing coronary flow reserve with PET

#### Image acquisition

Patients will be asked to abstain from methylxanthine derivatives, including caffeine, for ≥ 24 h before ^13^N-ammonia PET scan acquisition. Vasodilator medications such as nitrate, β-blockers or calcium channel blockers will be stopped 24 h before PET scan acquisition.

PET imaging will be performed using a Discovery 710 PET-CT scanner (GE Healthcare, Waukesha, WI, USA). An intravenous bolus injection of 550 MBq of ^13^N-ammonia will be given for both stress and rest image acquisition. A total of 47 transaxial slices will be reconstructed over an axial FOV of 15 cm. The acquired PET pixel size is 2.73 × 2.73 × 3.27 mm. A CT scout projection determines the heart position within the patient, and low-dose cine CT (100 kV, 10 mA, 0.5 second/rotation, 40-mm collimation, 5.5-second duration) will be used for attenuation correction of PET data. The PET acquisition consists of a series of dynamic scans from 0 to 6 minutes (12 × 10 seconds, 6 × 20 seconds and 2 × 60 seconds) followed by a single 20-minute gated static scan. Approximately 30 minutes after completion of rest imaging, stress imaging will be performed with adenosine (140 μg/kg/minute) infused intravenously for at least 3 minutes before tracer injection. CT-based attenuation correction will be repeated for the stress study.

#### Myocardial blood flow and coronary flow reserve

PET images will be reconstructed using an iterative ordered subsets expectation maximisation algorithm (2 iterations, 24 subsets), and absolute myocardial blood flow will be quantified with a commercially available software tool (SyngoMBF; Siemens Medical Solutions, Hoffman Estates, IL, USA). The software automatically determines the arterial input and myocardial uptake functions and calculates total and segmental perfusion using the model of DeGrado et al. [17]. Total and regional CFR of the three main coronary territories (left anterior descending, left circumflex and right coronary arteries) will be computed.

The above methodology will be used to assess CFR at baseline and after 24 weeks of therapy. The CFR is expected to increase following CXCR2 inhibition as compared with placebo.

### Procedures for assessing coronary restenosis and plaque composition

#### Image acquisition

Angiography and IVUS imaging will be performed after administration of 200 μg of intracoronary nitroglycerine. Angiography will be performed so that each vessel is viewed from at least two orthogonal angles after the planned PCI. For the IVUS procedure, a 45-MHz, 3.2-French, rotational IVUS catheter (Revolution; Volcano Corp.) will be used. After the IVUS catheter is placed in the distal vessel, the catheter will be pulled back to the aortic ostium using the motorised pull-back system at 0.5 cm/second. During pull-back, greyscale IVUS will be recorded and displayed in real time, and raw radiofrequency data will be captured at the top of the R wave for post-procedural reconstruction of the colour-coded map by a VH-IVUS data recorder (s5 Imaging System; Volcano Corp.).

#### Greyscale and VH-IVUS analyses

The smallest lumen at the area of percutaneous intervention is identified from axial and longitudinal plaque distribution. At this site, vessel cross‐sectional area (CSA) will be calculated. The degree of restenosis will be assessed at 24 weeks by IVUS to measure minimum luminal CSA. This will be measured during the baseline PCI and again following 24 weeks of therapy. The degree of restenosis is expected to be reduced after 24 weeks of treatment with CXCR2 inhibition.

Atherosclerotic coronary plaques are characterised by classification trees on the basis of mathematical autoregressive spectral analysis of IVUS back‐scatter data (echoPlaque 4; INDEC Systems, Los Altos, CA, USA). Fibrous areas are marked in green, fibrofatty in yellow, dense calcium in white and necrotic core in red on the reconstructed colour‐coded tissue map. The area and percentage area of each plaque component in the tissue map are calculated automatically using echoPlaque software.

Measurements are made for the entire length of vessel that VH-IVUS images are acquired. The volume of vessel and each plaque component is calculated using Simpson’s method and averaged over the length of the lesion to also generate a mean plaque area. Plaque composition will be assessed at baseline and after 24 weeks of therapy by IVUS using the Volcano Corp. system to obtain virtual histology. Each coronary artery will have the total plaque volume recorded and percentage volumes of fibrous, fibrofatty, necrotic core and calcific tissue. We will assess the change in necrotic core volume between baseline and after 24 weeks of therapy. The percentage volume of necrotic core is expected to be reduced after 24 weeks of treatment with CXCR2 inhibition as compared with placebo.

### Procedures for assessing invasive coronary physiology indices

Using specialised intracoronary wires (ComboWire) simultaneous measurements of coronary perfusion pressure and blood flow velocity will be performed that will provide specific coronary physiological data in addition to ^13^N-ammonia PET-determined CFR. With these high-fidelity measurements, we can estimate coronary flow, the components of the coronary wave profile and indices of microvascular function. During the invasive study, this wire will be placed in the distal coronary artery, and simultaneous measurements of aortic pressure, distal coronary pressure and distal coronary blood velocity will be performed during rest conditions and following adenosine (140 mg/kg/minute)-induced hyperaemia.

At the end of each study, data will be exported onto CD-ROMs, and appropriate cardiac cycles will be selected using custom-made software (Study Manager; Volcano Corp.). These selected beats will be analysed in another custom-made software application (Cardiac Waves, written in MATLAB, Delphi version 2010; Embarcadero Technologies, San Francisco, CA, USA). The flow and pressure data for the selected cardiac cycles are ensemble-averaged, which filters out background noise and allows identification and exclusion of non-physiological recordings. These provide means of average peak velocity (APV; the average of three to five instantaneous peak coronary flow velocities), aortic and distal coronary pressures, and wave intensity analysis (WIA) at each time point. These parameters will be used in calculations to determine the following indices:

1. *CFR*: We will assess APV at rest and following adenosine stress in the vessel that has undergone PCI. We can then determine vessel-specific regional CFR using the formula CFR = APV_stress_/APV_rest_.
2. *WIA*: This is a time-domain method of depicting a waveform in terms of a succession of multiple small wavefronts. WIA is invaluable in understanding the forces driving coronary flow, and a key element of this, the BEW, has been implicated in a number of disease processes [18]. We will assess WIA during conditions of adenosine-induced hyperaemia in all three vessels. We expect that there will be increased dominance of the BEW with CXCR2 therapy as compared with placebo.
3. *Microvascular resistance*: This is the ratio between distal coronary arterial pressure (P_d_) and coronary flow. The hyperaemic microvascular resistance (hMR) is a velocity-based index of microvascular resistance and is the ratio of P_d_ to APV during maximal hyperaemia. It is calculated using the formula hMR = P_d_/APV. Microvascular resistance is expected to be reduced after 24 weeks of treatment with CXCR2 inhibition as compared with placebo.

### Laboratory measurements

A full blood count will be performed by the haematology laboratory at St. Thomas’ Hospital. Blood (2 ml) will be sent in an ethylenediaminetetraacetic acid (EDTA) vacutainer tube to the laboratory within 30 minutes of venesection. Blood biochemistry (renal, liver, lipid, glycaemic and thyroid profiles), hs-CRP and homocysteine assays will be performed by the biochemistry laboratory at St. Thomas’ Hospital. Blood (10 ml) will be sent in a clotted vacutainer tube to the laboratory within 30 minutes of venesection.

Circulating MPA measurement will be performed in our research laboratories by flow cytometric analysis of whole blood (4 ml) collected in sodium citrate (0.3% final concentration). Immediately after venepuncture, blood will be immunostained with different combinations of peridinin–chlorophyll-protein complex-conjugated anti-human CD14, fluorescein isothiocyanate-conjugated anti-human CD16 and allophycocyanin-conjugated anti-human CD42b or CD62P. Isotype control antibodies will be used as negative controls. After red cell lysis using fluorescence-activated cell sorting lysing solution (BD Biosciences, San Jose, CA, USA), samples will be fixed in 1% paraformaldehyde and kept at 4 °C until analysed within 48 h by flow cytometry.

At visits 4, 8 and 11, we will measure pre-dose (trough) and peak (2 h post-dose) AZD5069 plasma concentrations in all subjects. Pharmacokinetic assays of AZD5069 and its major human metabolite AZ13587715 will be conducted via a contracted vendor (Covance Inc., Princeton, NJ, USA) that has created and validated the LC/MS-based analytical method for assessment of AZD5069 and AZ13587715 in human plasma preserved in EDTA and stored between − 10 °C and − 30 °C. The analytical method will be identical to that used in prior human studies of AZD5069 [13, 19–21]. At monthly intervals, spot urine samples will be obtained from women of childbearing potential in order to exclude pregnancy (Alere hCG Easy; Alere Inc., Waltham, MA, USA).

### Emergency code break

A 24-h emergency code break and medical information will be provided by ESMS. Each randomised subject will be provided with a card detailing code break telephone numbers and emergency contact details. Subjects will be requested to carry this card with them always whilst participating in the trial.

### Withdrawal of subjects

The study drug must be discontinued if any of the following occur:

- An exclusion criterion is incident during the study.
- Absolute neutrophil count < 1 10^9^/L is noted in two consecutive samples within 48 h.
- The participant misses > 20% of his or her doses.
- The participant decides he or she no longer wishes to continue.
- It is recommended by the investigator.

Participants have the right to withdraw from the study at any time for any reason. The investigator also has the right to withdraw patients from the study drug in the event of inter-current illness, adverse events (AEs), serious adverse events (SAEs), suspected unexpected serious adverse reactions (SUSARs), protocol violations, cure, or administrative or other reasons. It is understood by all concerned that an excessive rate of withdrawals can render the study un-interpretable; therefore, unnecessary withdrawal of patients should be avoided. Should a patient decide to withdraw from the study, all efforts will be made to report the reason for withdrawal as thoroughly as possible. Should a patient withdraw from study drug only, efforts will be made to continue to obtain follow-up data with the permission of the patient. Participants who wish to withdraw from trial medication will be asked to confirm whether they are still willing to provide the following:

- Trial-specific data at subsequent scheduled visits
- Clinical follow-up data collected as per routine clinical practice

### Reporting procedure for all adverse events

Information on all AEs, serious and non-serious, is collected, documented and reported on the appropriate eCRFs/SAE reporting forms once informed consent has been signed, and this will end 14 days after completing the study medication. The following information is recorded:

- Participant details
- AE description
- Body system code
- Start date of event
- End date of event
- Outcome of event
- Intensity/severity of event
- Study procedure related (i.e., causality/relatedness)
- Investigational Medical Product (IMP) related (i.e., causality/relatedness)
- Whether the event is serious
- Follow-up information recorded as necessary

AEs considered to be related to study procedure or medication as judged by a medically qualified investigator are followed until resolution or the event is considered stable. All related AEs that result in a subject’s withdrawal from the study or present at the end of the study are followed until a satisfactory resolution occurs.

### Reporting procedure for serious adverse events

King’s Health Partners Clinical Trials Office (KHP-CTO) will have responsibility for pharmacovigilance. All SAEs, SARs and SUSARs will be reported immediately by the chief investigator (and certainly no later than 24 h) to the KHP-CTO in accordance with the current pharmacovigilance policy. The chief investigator will report SUSARs to the ethics committee.

### Statistics and sample size calculation

Because the trial is randomised and double-blinded, all blood assays, ^13^N-ammonia PET scans and invasive coronary assessments will be performed with no knowledge of subject treatment allocation (AZD5069 or placebo). The primary outcome is change in total CFR as measured by ^13^N-ammonia PET. Formal power calculations are problematic because no data exist to date in humans on the relationship between CXCR2 inhibition and CFR. However, assuming a medium to large effect size (*d* = 0.7), α error probability of 0.05 (two-tailed), power 0.80 and allocation ratio 2:1 (active drug to placebo), power calculation (using G*Power 3.1.3 software) indicates that we would need 51 subjects in the active and 25 subjects in the placebo group.

Furthermore, unpublished observational data from AstraZeneca and the Göteborg Hospital (Göteborg, Sweden) which relate neutrophil counts in stable CAD and after myocardial infarction with echocardiographic CFR are shown in Table 1. On the basis of these analyses, we will recruit 60 into the active group and 30 into the placebo group, firstly to allow for lesser effect sizes and secondly to allow us to better stratify responses to CXCR2 inhibition on the basis of plasma IMP levels achieved.

**Table 1:** Relationship between neutrophil count and coronary flow reserve based on previous studies

Study	Neutrophil count	Echo-based CFR (mean ± SE)	CFR increase	SD of PET-MPR [22]	Patients required (*p* < 0.05, 80% power)
Stable CAD cohort					
	5.8	2.10 ± 0.19		0.36	
	4.6	2.35 ± 0.12	10.6%	0.36	33
	4.2	2.43 ± 0.11	13.5%	0.36	19
Post-MI cohort					
	5.8	2.45 ± 0.07		0.36	
	4.6	2.64 ± 0.05	7.2%	0.36	57
	4.2	2.70 ± 0.05	9.3%	0.36	33

*Abbreviations: CFR* Coronary flow reserve, *PET* Positron emission tomography, *MPR* Myocardial perfusion reserve, *CAD* Coronary artery disease, *MI* Myocardial infarction

The relationship between neutrophil count and echo-based CFR in both stable CAD and post-MI cohorts is shown. This demonstrates that lower neutrophil counts are associated with higher CFR

A secondary outcome is the change in the magnitude of the BEW, a key component of the coronary wave profile, with maximal hyperaemia. From previous work, we expect the magnitude of the BEW to increase 40% with adenosine induced maximal hyperaemia in patients with CAD. Unpublished data from the host centre suggest that in patients without CAD, the BEW can increase a further 14%. Assuming an α error probability of 0.05 (two-tailed), power 0.80 and allocation ratio 2:1 (active drug to placebo), power calculation (using G*Power 3.1.3 software) indicates that we would need 26 subjects in the active and 13 subjects in the placebo group. The BEW measurement has a standard deviation of 21%, as such we will need to recruit 57 patients in total to undergo ComboWire assessment as part of their invasive protocol.

A full description of the statistical analysis plan is beyond the scope of this protocol, and will be published separately. In summary, the primary analysis will be an intention to treat analysis. An analysis of covariance model will be used to obtain an estimate for the mean difference in total CFR between the two treatment groups including covariates for baseline CFR. The estimated treatment effect will be reported with 95% confidence intervals and corresponding *p* value.

### Data collection

#### Electronic case report form

Data are captured in an eCRF which is provided, maintained and stored by KHP-CTO in London, UK. The eCRFs are a component of the custom-designed, trial-specific database built using the InferMed MACRO application, version 4.0 (InferMed/Elsevier, London, UK). Clinical data for the trial are generated by clinical assessments, diagnostic tests, laboratory tests and interviews with the patient during study visits. Source data take the form of patient medical records, hospital records, hospital letters/communications, laboratory reports and so forth. These data are entered into the MACRO application by research staff at the trial site associated with the CICADA study. Privacy of the patients is guaranteed; stored data and materials will be identifiable to the person only by a sequentially assigned subject number. The eCRF is designed in accordance with the requirements of the study protocol and complies with regulatory requirements. Access to the eCRF is password-protected, and the password is given only to site personnel. Data generated throughout the study are monitored, and the eCRFs are checked against the subject records for accuracy. Following completion of the eCRFs, the data are checked electronically for consistency and plausibility by pre-defined range checks. If necessary, automatic queries are generated for questionable data.

#### Data quality control

The clinical research associate and the investigators meet frequently to review study progress and procedures and to discuss any AEs or dropouts. Furthermore, a data monitoring committee has been established to safeguard the interests of the trial’s participants and to monitor the data collected in the trial. The data monitoring committee will meet prior to the start of the study and 6–12 months after the start of the study. The data monitoring committee may also meet on an ad hoc basis should the need arise. The data collection, management, analysis and interpretation, as well as production of publications, are independent of the funding bodies and other competing interests. The trial results will be disseminated via journal publication and conference presentation without exposing the identity of the trial subjects.

### Monitoring

Monitoring is undertaken per good clinical practice guidelines and the study monitoring plan. The study monitor is suitably trained, qualified and experienced to perform this task. Data are evaluated for compliance with the protocol and accuracy in relation to source documents. The following data are assessed:

- Written informed consent
- Flowchart filled in for included and excluded subjects
- Trial progress
- Primary and secondary outcome collection
- SAEs
- Drug accountability of the study treatment

## Discussion

Atherosclerosis is the primary cause of mortality and morbidity in the Western world. Its pathophysiology is not fully understood, but both hyperlipidaemia and inflammation represent central pillars to both atherogenesis and plaque destabilisation. Recent advances point to a contributory role of neutrophils during this process, which offers a novel therapeutic target that could improve outcomes in patients with CAD.

Our study is focused on cardiovascular surrogate parameters, such as CFR function, which cannot replace outcome trials but can provide insights into the potential mechanisms of the cardiovascular effects of CXCR2 inhibition. Moreover, currently ongoing trials are not specifically focused on modulation of neutrophil activity as a target of intervention. We therefore believe that our study will contribute to a better understanding of the role of neutrophil-mediated inflammation in CAD.

## Trial status

Recruitment is ongoing.

## Additional file

Additional file 1: SPIRIT 2013 checklist: recommended items to address in a clinical trial protocol and related documents. (DOC 121 kb)

## Abbreviations

AE: Adverse event
APV: Average peak velocity
ARSAC: Administration of Radioactive Substances Advisory Committee
BEW: Backward expansion wave
CAD: Coronary artery disease
CICADA: CXCR2 Inhibition – a novel approach to treating CoronAry heart DiseAse
CFR: Coronary flow reserve
CRP: C-reactive protein
CSA: Cross-sectional area
CT: Computed tomography
CXCL: Chemokine (C-X-C motif) ligand
CXCR2: Cysteine-X-cysteine chemokine receptor 2
ECG: Electrocardiogram
eCRF: Electronic case report form
EDTA: Ethylenediaminetetraacetic acid
ESMS: Emergency Scientific and Medical Services
hMR: Hyperaemic microvascular resistance
hs-CRP: High-sensitivity C-reactive protein
IL-8: Interleukin-8
IVUS: Intravascular ultrasound
KHP-CTO: King’s Health Partners Clinical Trials Office
LDL: Low-density lipoprotein
LPS: Lipopolysaccharide
MHRA: Medicines and Healthcare products Regulatory Agency
MI: Myocardial infarction
MPA: Monocyte-platelet aggregate
MPR: Myocardial perfusion reserve
PCI: Percutaneous coronary intervention
P_d_: Distal coronary arterial pressure
PET: Positron emission tomography
SAE: Serious adverse event
SPIRIT: Standard Protocol Items: Recommendations for Interventional Trials
SUSAR: Suspected unexpected serious adverse reaction
VH-IVUS: Virtual histology intravascular ultrasound
WIA: Wave intensity analysis
